# Ultrasound intima media thickness cut-off values for cranial and extracranial arteries in patients with suspected giant cell arteritis

**DOI:** 10.3389/fmed.2022.981804

**Published:** 2022-08-26

**Authors:** Katerine López-Gloria, Isabel Castrejón, Juan Carlos Nieto-González, Pablo Rodríguez-Merlos, Belén Serrano-Benavente, Carlos Manuel González, Indalecio Monteagudo Sáez, Teresa González, José María Álvaro-Gracia, Juan Molina-Collada

**Affiliations:** ^1^Department of Rheumatology, Hospital General Universitario Gregorio Marañón, Madrid, Spain; ^2^Instituto de Investigación Sanitaria Gregorio Marañón (IiSGM), Madrid, Spain

**Keywords:** ultrasound, giant cell (temporal) arteritis, vasculitis, imaging, arteries

## Abstract

**Objective:**

To determine the optimal ultrasound (US) cut-off values for cranial and extracranial arteries intima media thickness (IMT) to discriminate between patients with and without giant cell arteritis (GCA).

**Methods:**

Retrospective observational study including patients referred to an US fast-track clinic. All patients underwent bilateral US examination of the cranial and extracranial arteries including the IMT measurement. Clinical confirmation of GCA after 6 months was considered the gold standard for diagnosis. A receiver operating characteristic (ROC) analysis was performed to select the cut-off values on the basis of the best tradeoff values between sensitivity and specificity.

**Results:**

A total of 157 patients were included, 47 (29.9%) with clinical confirmation of GCA after 6 months. 41 (87.2%) of patients with GCA had positive US findings (61.7% had cranial and 44.7% extracranial involvement). The best threshold IMT values were 0.44 mm for the common temporal artery; 0.34 mm for the frontal branch; 0.36 mm for the parietal branch; 1.1 mm for the carotid artery and 1 mm for the subclavian and axillary arteries. The areas under the ROC curves were greater for axillary arteries 0.996 (95% CI 0.991–1), for parietal branch 0.991 (95% CI 0.980–1), for subclavian 0.990 (95% CI 0.979–1), for frontal branch 0.989 (95% CI 0.976–1), for common temporal artery 0.984 (95% CI 0.959–1) and for common carotid arteries 0.977 (95% CI 0.961–0.993).

**Conclusion:**

IMT cut-off values have been identified for each artery. These proposed IMT cut-off values may help to improve the diagnostic accuracy of US in clinical practice.

## Introduction

Giant cell arteritis (GCA) is the most frequent systemic vasculitis in elderly patients. An early diagnosis and prompt treatment is crucial to prevent serious complications (1–3). Ultrasound (US) is a valid and reliable tool to detect inflammation in patients with GCA, the non-compressible halo sign being the most relevant US finding. It has been defined by the US Outcome Measures in Rheumatology (OMERACT) large vessel vasculitis working group as a “homogenous, hypoechoic wall thickening that is well delineated toward the luminal side that is visible both in longitudinal and transverse planes, most commonly concentric in transverse scans” (4). According to recent EULAR recommendations, patients with high clinical suspicion of GCA and a positive imaging test do not need additional tests, such as biopsy or other imaging methods, for GCA diagnosis (5). However, false-positive halos may be present in other forms of vasculitis (e.g., in ANCA-associated vasculitis), amyloidosis (6), infectious diseases or even in patients with atherosclerosis (7, 8). Thus, a positive halo needs to be interpreted in the clinical context with an evaluation of the pretest probability (9).

Although the halo sign has been considered the most useful sign to support GCA diagnosis, it can be highly influenced by the sonographer skills and the quality of the US equipment for Doppler settings and artifacts. Sensitivity is highly variable between studies, showing a pooled sensitivity of 77% and a pooled specificity of 96% compared to the clinical diagnosis of GCA (10). Nowadays, thanks to the availability of high resolution transducers, intima media thickness (IMT) of cranial and extracranial arteries can be accurately measured as homogeneous, hypoechoic or anechoic structure delineated by two parallel hyperechoic margins (11). An increased IMT suggestive of GCA may be easily assessed by US, giving the classical appearance of a halo sign using color Doppler mode. However, few data are published regarding the optimal cut-off values for IMT to differentiate patients and controls in clinical practice (12, 13), and only one group studied the subclavian and common carotid arteries, which should require replication.

Our primary objective is to define cut-off values for cranial and extracranial arteries to discriminate patients with and without GCA.

## Materials and methods

### Patients

This is a retrospective observational study including patients referred to a US fast-track pathway (14) for screening of possible GCA from 2019 to 2021. In our academic center, patients with suspected GCA are referred for US examination within 24 h per protocol. A bilateral US exam of the cranial (superficial temporal arteries and its frontal and parietal branches) and extracranial (carotid, subclavian, and axillary arteries) arteries is performed as part of the diagnostic work-up. The following inclusion criteria were applied: patients age > 18 years with GCA suspicion according to clinician criteria (history of ESR > 20 mm/h or CRP > 5 mg/l, or cranial/extracranial symptoms of GCA or PMR symptoms). Patients with a previous diagnosis or clinical history of GCA were excluded. All exams were performed in routine daily practice conditions including consecutive patients.

### Data collection

The following variables were collected: demographics, presenting symptoms, previous use of glucocorticoids or polymyalgia diagnosis, and laboratory variables as C- reactive protein (CRP), erythrocyte sedimentation rate (ESR), hemoglobin and platelets. GCA clinical diagnosis after 6 months follow-up by the referring rheumatologist was the gold standard for diagnosis.

### Ultrasound assessment

All patients underwent bilateral US examination of the three temporal artery (TA) segments (common superficial TA, it parietal and frontal branches) and extracranial (common carotid, subclavian and axillary) arteries within 24 h per protocol (excluding weekends with delays up to 48 h). The exam was performed in a supine position, by the same evaluator (JMC) using an EsaoteMyLab8 (Esaote, Genoa). For superficial TA at it parietal and frontal branches we used a 12–18 MHz frequency transducer [B-mode frequency, 18 MHz; depth, 15 mm; focus point at approximately 3–5 mm below the skin surface, depending on the depth of the segment; color Doppler frequency, 11 MHz, pulse repetition frequency (PRF), 2.0 KHz]. An 8–14 frequency transducer was used for extracranial arteries (B-mode frequency, 14 MHz; depth, 3 cm; focus at 2 cm below the skin surface depending on the depth of the segment; color Doppler frequency, 9 MHz; PRF, 3.0 kHz). The subclavian arteries were scanned at the infraclavicular fossa and axillary arteries at the axillary fossa. The IMT was measured in gray scale mode and the presence of a non-compressible halo sign was checked in all arteries. We checked the presence of a halo defined according to the recent OMERACT Large Vessel Vasculitis Ultrasound Working Group definition (4). The measurement of the IMT of each artery was made from the luminal–intimal interface to the medial–adventitial interfaces on the arterial wall distal to the probe on longitudinal planes. The measurement of the IMT was obtained on B mode in all arteries (not only those showing a halo). For the frontal and parietal branches, the IMT was measured approximately 1 cm distal to the bifurcation of the superficial TA, and for axillary arteries at the level of the humeral head. Color Doppler signal was used to confirm the correct measurement of the IMT in doubtful cases. The presence of a halo and/or compression sign in temporal arteries and the presence of a halo in extracranial arteries was considered a positive US finding for GCA.

### Statistical analysis

Quantitative data were described as mean (S.D.) and qualitative variables as absolute frequency and/or corresponding percentages. Mean IMT values of each artery were compared between patients according to GCA clinical diagnosis by independent samples *T*-test. Receiver operating characteristics (ROC) analysis was performed and the Youden index was used to determine the optimal cut-off value for IMT of each artery. SPSS software (version 23.0; IBM, Armonk, NY, United States) was used for statistical analysis.

### Ethical approval

This study was performed in accordance with the ethical standards of the responsible committee on human experimentation and the Helsinki Declaration of 1975, as revised in 1983. Research ethics committee approval for the protocol was obtained prior to commencing the study (RHEUM0322) and written informed consent was determined to be not mandatory.

## Results

### Patients’ characteristics

A total of 157 patients evaluated in the US fast-track pathway were included for analysis, of whom 67.5% were female and mean (*SD*) age was 73.7 (10.8) years, median (interquartile range 25th–75th) 74 (66–82). Polymyalgia rheumatica diagnosis before US examination was present in 43 (27.4%) patients and 78 (50%) patients were on steroids before US examination, mean (*SD*) corticosteroid dose was 48.4 (146.5) mg/day (min 2.5, max 1,000). After 6-months of follow-up, 47 (29.9%) patients had GCA clinical confirmation according to clinician diagnosis. TA biopsy was performed per clinician criteria in 31 patients, 10 (43.5%) with positive results. Clinical, laboratory and main US findings of patients with and without GCA are shown in Table 1. GCA patients had higher acute phase reactants: CRP [mg/dL) 10.7 (18.2) vs. 3.8 (5), *p* = 0.001 and ESR (mm/h) (68.2 (34) vs. 45 (31.8), *p* = 0.001].

**TABLE 1:** Clinical, laboratory and US findings of patients with and without GCA.

	Total *n* = 157	Patients with GCA *n* = 47 (29.9)	Patients without GCA *n* = 110 (70.1)	*P*
**Demographics**				
Age, mean (*SD*)	73.7 (10.8)	75.3 (11.3)	73 (10.6)	0.245
Female, *n* (%)	106 (67.5)	31 (66)	75 (68.2)	0.785
Arterial hypertension, *n* (%)	96 (61.5)	30 (65.2)	66 (60)	0.541
Mellitus diabetes, *n* (%)	37 (23.7)	13 (28.3)	24 (21.8)	0.388
Smoker, *n* (%)	16 (10.3)	6 (13)	10 (9.1)	0.458
Former smoker, *n* (%)	26 (16.7)	10 (21.7)	16 (14.5)	0.272
**Clinical variables**				
Baseline use of steroids, *n* (%)	78 (50)	21 (45.7)	57 (51.8)	0.482
PMR diagnosis before US examination, *n* (%)	43 (27.4)	8 (17)	35 (31,8)	0.121
**Laboratory findings**				
CRP (mg/dL), mean (*SD*)	5.9 (11.3)	10.7 (18.2)	3.8 (5)	0.001
ESR (mm/h), mean (*SD*)	52.8 (34.2)	68.2 (34)	45 (31.8)	0.001
Hemoglobin (g/dL), mean (*SD*)	13.7 (17.6)	11.7 (1.6)	14.5 (21)	0.185
Platelets 10^9^/L, mean (*SD*)	293 (124.7)	335.4 (143.3)	274.7 (111.6)	0.014
**US variables**				
Positive US findings*, *n* (%)	46 (29.3)	41 (87.2)	5 (4.5)	<0.001
Temporal artery positive US findings, *n* (%)	32 (20.4)	29 (61.7)	3 (2.7)	<0.001
Extracranial arteries positive US findings, *n* (%)	23 (14.6)	21 (44.7)	2 (1.8)	<0.001
Temporal + extracranial arteries positive US findings, *n* (%)	9 (5.7)	9 (19.1)	0 (0)	<0.001

PMR, polymyalgia rheumatica; CRP, C-reactive protein; ESR, erythrocyte sedimentation rate; US, ultrasound; SD, standard deviation; *Presence of a halo and/or compression sign in temporal arteries and/or presence of a halo in extracranial arteries.

### Ultrasound findings

**FIGURE 1:**
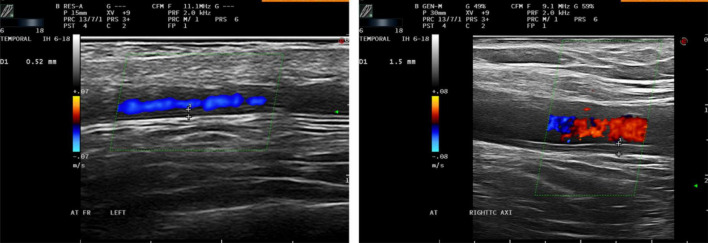
Longitudinal US scan (left) of the frontal branch of the left temporal artery in a patient with newly GCA diagnosis. Longitudinal US scan (right) of the right axillary artery in patient with large vessel GCA. Both arteries show increase IMT above the proposed cut-off values.

US findings are presented in detail in Tables 1, 2. In total, 41 (87.2%) patients with GCA and only 5 (4.5%) patients without GCA had positive US findings (overall sensitivity 87.2%, specificity 95.5%, positive predictive value 89.1%, negative predictive value 94.6%). Among patients with GCA, the most frequent finding was temporal artery involvement in 29 (61.7%) patients, followed by extracranial involvement in 21 (44.7%) patients and 9 (19.1%) patients with a mixed pattern of cranial and extracranial arteries involvement (Figure 1).

**TABLE 2:** Optimal IMT cut-off values for cranial and extracranial arteries.

Artery	Side	Patients without GCA	Patients with GCA	Cut-off (mm)	AUC (CI 95%)	Sensitivity (%)	Specificity (%)	Geometric mean
Common superficial temporal artery mm, mean (*SD*)	Right	0.33 (0.06)	0.68 (0.28)	0.43	0.997 (0.988–1)	100	97.1	0.985
	Left	0.35 (0.11)	0.57 (0.21)	0.45	0.966 (0.905–1)	100	92.3	0.961
	Both	0.34 (0.08)	0.63 (0.25)	0.44	0.984 (0.959–1)	94.7	95.1	0.949
Frontal branch mm, mean (*SD*)	Right	0.26 (0.05)	0.4 (0.18)	0.34	0.994 (0.983–1)	100	97.1	0.985
	Left	0.27 (0.05)	0.4 (0.18)	0.34	0.985 (0.962–1)	100	96.1	0.980
	Both	0.26 (0.05)	0.4 (0.18)	0.34	0.989 (0.976–1)	100	96.6	0.983
Parietal branch mm, mean (*SD*)	Right	0.27 (0.05)	0.43 (0.18)	0.36	0.994 (0.981–1)	100	98.9	0.994
	Left	0.27 (0.05)	0.41 (0.16)	0.36	0.987 (0.967–1)	100	97.6	0.988
	Both	0.27 (0.05)	0.42 (0.17)	0.36	0.991 (0.980–1)	100	98.3	0.991
Carotid mm, mean (*SD*)	Right	0.8 (0.17)	0.88 (0.29)	1	0.974 (0.949–0.999)	100	92.6	0.962
	Left	0.82 (0.15)	1 (0.42)	1.2	0.982 (0.961–1)	90.9	96.2	0.935
	Both	0.81 (0.16)	0.96 (0.36)	1.1	0.977 (0.961–0.993)	90	94	0.920
Subclavian mm, mean (*SD*)	Right	0.74 (0.18)	0.99 (0.44)	1	0.987 (0.97–1)	100	93.4	0.966
	Left	0.67 (0.17)	0.9 (0.35)	1.1	0.991 (0.975–1)	100	98.3	0.991
	Both	0.7 (0.18)	0.94 (0.4)	1	0.99 (0.979–1)	100	96	0.980
Axillary mm, mean (*SD*)	Right	0.69 (0.16)	0.99 (0.5)	1	0.992 (0.982–1)	100	96	0.980
	Left	0.67 (0.17)	0.99 (0.49)	1	0.998 (0.995–1)	100	98.3	0.991
	Both	0.68 (0.17)	0.99 (0.49)	1	0.996 (0.991–1)	100	97.1	0.985

### Intima media thickness cut-off values

Values of IMT of cranial and extracranial arteries in patients with and without GCA are shown in Table 2. The IMT cut-off values showing the highest diagnostic accuracy to discriminate between patients with and without GCA were for the cranial arteries (0.44 mm for the common superficial temporal artery; 0.34 mm for the frontal branch and 0.36 mm for the parietal branch) and for the extracranial arteries (1.1 mm for the carotid artery and 1 mm for the subclavian and axillary arteries) (Table 2). The area under the ROC curve of the IMT for a clinical diagnosis of GCA was 0.984 (95% CI 0.959–1) for common superficial temporal artery, 0.989 (95% CI 0.976–1) for frontal branch, 0.991 (95% CI 0.980–1) for parietal branch, 0.977 (95% CI 0.961–0.993) for carotid, 0.99 (95% CI 0.979–1) for subclavian and 0.996 (95% CI 0.991–1) for axillary arteries. Sensitivities and specificities of each IMT cut-off value are shown in Table 2.

## Discussion

Few studies have defined IMT cut-off values for GCA diagnosis after adequate evaluation. In this observational cross-sectional study, we provide the optimal cut-off values for IMT of cranial and extracranial arteries.

Several studies have shown the usefulness and good performance of US for the diagnosis of GCA (15–19) and this has led to the recent EULAR recommendations (5) identifying US as the test of initial imaging in patients with suspected GCA presenting with predominantly cranial symptoms. Although the halo sign is considered the most characteristic US finding of GCA, the measurement of IMT provides a more accurate diagnosis, probably because it is not influenced by potential Doppler artifacts. However, when analyzing a quantitative measure as a diagnostic test it is important to provide the optimal cut-off values to differentiate between patients and controls.

In our sample, 87.2% of patients with GCA had positive US results with a positive predictive value of 89.1%. The most frequent finding was the involvement of the temporal arteries (61.7% of patients), followed by extracranial involvement (44.7%). The area under the ROC curve of each explored artery were high with excellent sensitivities and specificities and proposed IMT cut-off values showed the highest diagnostic accuracy to discriminate between patients and controls. These values are high enough to justify the use of these cut-off values with high diagnostic precision. Previous studies have suggested variables cut-off values for temporal arteries: 0.3 mm (20), 0.4 mm (21), 0.5 mm (22), 0.7 mm (23), and 1 mm (24). Moreover, cut-off values for extracranial arteries of 1.3 mm (25) and 2 mm (26), and 1.5 mm (27) for the axillary artery exclusively, have been suggested. All of them based on the criteria and clinical experience of each author, respectively. Our results are in line with a recent study by Ješe et al. (12), in which they determined potential cut-off values for IMT in seven preselected arteries (temporal, facial, occipital, carotid, vertebral, subclavian, and axillary) comparing them between patients with and without GCA. They found positive US findings in 98.4% of patients with GCA with involvement of the temporal artery in 77.4%, and involvement of extracranial arteries in 35.1%. In relation to the cut-off values, they reached high levels of diagnostic precision with an IMT of 0.4 mm for the temporal arteries and an IMT of 1 mm for the carotid, subclavian and axillary arteries. Our findings were also compared with a recent study investigating IMT cut-off values by Schäfer et al. (13) who compared the IMT of 40 newly diagnosed GCA patients and 40 healthy controls. In this study the control group consisted in patients with other rheumatic and non-rheumatic diseases, but not suspected GCA. They established cut-off values of 0.42 mm for the common superficial temporal artery, 0.34 mm for the frontal branch, 0.29 mm for the parietal branch, and 1 mm for the axillary artery. Our cut-off values for IMT of 0.44 mm for the common superficial temporal artery and 0.36 mm for the parietal branch differ from those proposed by Schäfer et al. (0.42 for the common superficial temporal artery and 0.29 for the parietal branch). Although we used a 18 MHz probe, these slight differences should be addressed in further studies. The proposed IMT cut-off of 1 mm for the axillary artery coincides with previous studies (13, 28). Our group previously presented preliminary data on the diagnostic value of IMT cut-off values studying the same population included in this work, with similar results (29).

Examination of the axillary arteries in patients with suspected GCA is most useful when US of the temporal arteries is negative or inconclusive and there is a high clinical suspicion of large vessel GCA (5). Although in this scenario it is not clear which extracranial arteries should be examined (30). Skoog et al. (31) recently evaluated the diagnostic performance of an extended US protocol (which in addition to temporal and axillary arteries, also includes subclavian, brachiocephalic, and carotid arteries) in patients with suspected GCA. According to their results, they found that 86% had involvement of the temporal arteries and 28% of patients showed inflammatory changes in both the temporal and extracranial arteries with a sensitivity of 95% and specificity of 98%. In our population, the sensitivity and specificity were slightly lower with values of 87.2 and 95.5%, respectively.

In 2018, De Miguel et al. (8) evaluated 40 patients with high cardiovascular risk and found an association between atherosclerotic disease and an increase of IMT values of the temporal arteries. Taking into account that atherosclerosis is prevalent in this group of patients presenting with clinical symptoms of GCA, they proposed a cut-off value for IMT of 0.34 mm in at least two temporal artery branches. In our study, the cut-off value for IMT of the temporal artery and its branches are in accordance with that proposed by De Miguel although the cardiovascular risk in our population was not specifically investigated. Despite the availability of excellent IMT cut-off values for the cranial and extracranial arteries, it is always important to take into account the clinical context of the patient, since findings above the proposed IMT cut-off values may be present in patients with atherosclerosis.

The main strength of our study is the systematically assessment of IMT in patients with suspected GCA in a well-established fast-track clinic. Our study has limitations including the retrospective design and being conducted in a single center. The initial use of steroids was relatively high, in almost half of the patients, that may affect the sensitivity of the US examination. However, polymyalgia rheumatica before US was present in 27.4% of the study population, finding comparable to previous studies (1, 32, 33). Ultrasonographer was not blinded to clinical data and clinician’s making the diagnosis were not blinded to US findings, that may lead to bias in the diagnostic accuracy of US, although this bias is common in all studies aiming at validate a diagnostic tool in GCA. On the other hand, since no gold standard is valid for GCA diagnosis, we selected the clinical diagnosis at 6-month as the reference standard. However, as the majority of patients may continue with corticosteroid treatment at this time, other potential diseases may be masked. In addition, inter-observer reliability was not be investigated.

In summary, US of cranial and extracranial arteries has shown great diagnostic performance as an initial diagnostic test in patients with suspected GCA. These proposed cut-off values for IMT may help to improve the diagnostic accuracy of US in clinical practice. Finally, further validation of the cut-off values in different cohorts will be needed.

## Data availability statement

The original contributions presented in this study are included in the article/supplementary material, further inquiries can be directed to the corresponding author.

## Ethics statement

The studies involving human participants were reviewed and approved by Research Ethical Committee of Hospital General Universitario Gregorio Marañón (RHEUM0322). Written informed consent for participation was not required for this study in accordance with the national legislation and the institutional requirements.

## Author contributions

KL-G and JM-C performed the study design, statistical analysis, subject recruitment, US examinations, and collection of the epidemiological and clinical data. KL-G, IC, JN-G, PR-M, BS-B, CG, IM, TG, JÁ-G, and JM-C drafted the manuscript. All authors revised the final manuscript and made substantial contributions to the conception and design of this study.
